# Curcumin Loaded ZIF 8 Nanomaterials Ameliorate Porcine Endometritis Like Lesions in a Mouse Model via Regulating Macrophage Polarization and Antibacterial Activity

**DOI:** 10.3390/ani16182964

**Published:** 2026-09-20

**Authors:** Ruijing Su, Shuqin Fang, Xinhong Li, Tianlong Liu, Hongxiu Diao

**Affiliations:** 1 Key Laboratory of Animal Pathogen Infection and Immunology of Fujian Province, College of Animal Sciences, Fujian Agriculture and Forestry University, Fuzhou 350002, China; suruijing@fafu.edu.cn (R.S.);; 2 Key Laboratory of Fujian-Taiwan Animal Pathogen Biology, College of Animal Sciences, Fujian Agriculture and Forestry University, Fuzhou 350002, China; 3 Joint Laboratory of Animal Pathogen Prevention and Control of Fujian-Nepal, College of Animal Sciences, Fujian Agriculture and Forestry University, Fuzhou 350002, China; 4 National Key Laboratory of Veterinary Public Health and Safety, College of Veterinary Medicine, China Agricultural University, No. 2 Yuanmingyuan West Road, Beijing 100193, China

**Keywords:** porcine endometritis, curcumin-loaded ZIF-8, dominant pathogenic bacteria, antibacterial and anti-inflammatory, macrophage polarization

## Abstract

Porcine endometritis, primarily resulting from mixed bacterial infections, adversely impacts sow reproductive performance and leads to substantial economic losses in the pig farming sector. Traditional antibiotic treatment is limited due to bacterial resistance and drug residue issues. Curcumin (Cur) possesses inherent antibacterial and anti-inflammatory properties; however, its use is restricted due to limited water solubility and low bioavailability. In this work, dominant pathogens were isolated and identified from vaginal swabs collected from 48 sows with clinical endometritis via 16S rRNA sequencing, followed by antibiotic susceptibility assessment. Cur-loaded ZIF-8 nanoparticles (Cur@ZIF-8) were synthesized for non-antibiotic therapeutic exploration against porcine endometritis. Physicochemical characterizations revealed that Cur@ZIF-8 possessed a hydrodynamic particle size of 106.2 nm, a curcumin loading efficiency of 10.8%, and an encapsulation efficiency of 86.4%. The nanoparticles exhibited uniform morphology, favorable dispersibility, and showed a moderate increase in curcumin release under acidic conditions. Hemolysis assays and mouse toxicity tests confirmed the favorable biocompatibility of Cur@ZIF-8 without obvious toxic effects. In vitro antibacterial evaluations demonstrated that Cur@ZIF-8 exerted potent bactericidal effects, with the minimum inhibitory concentration (MIC) against *Proteus mirabilis* determined to be 12.5 μg/mL. Consistent with clinical findings, *Proteus mirabilis*, *Staphylococcus aureus*, and *Escherichia coli* were confirmed as the major causative agents of porcine endometritis, which frequently occurred as mixed infections, and these clinical isolates exhibited prominent multi-drug resistance to common antibiotics. Using the isolated dominant clinical strains, we established mouse endometritis models and performed in vivo therapeutic evaluations via uterine bacterial load enumeration, histopathological observation, and immunofluorescence detection of macrophage polarization. The results demonstrated that Cur@ZIF-8 markedly mitigated uterine pathological injury, reduced intrauterine bacterial burden, and alleviated inflammatory infiltration. A decreased semi-quantitative histopathological inflammation score was observed in uterine tissues after Cur@ZIF-8 intervention. Mechanistically, Cur@ZIF-8 remodels the uterine inflammatory microenvironment through the modulation of macrophage polarization, achieving combined antibacterial and anti-inflammatory functions. Collectively, in vivo experiments in mouse endometritis models verified that the Cur@ZIF-8 nanoplatform produces antibacterial and anti-inflammatory effects and exhibits excellent biosafety and biocompatibility. This preclinical study offers a promising non-antibiotic nanotherapeutic candidate and provides an alternative strategy to reduce antibiotic consumption and combat antimicrobial resistance in livestock production; nevertheless, further systematic validation in sow models is still needed to support its practical clinical application for porcine endometritis control.

## Simple Summary

Sow uterine inflammation is mostly triggered by multiple bacteria, which damages sows’ breeding ability and causes heavy financial losses for pig farms. Common antibiotic treatments are becoming less effective because bacteria can develop drug resistance, and leftover medicine may also bring safety risks. A natural substance called curcumin can fight bacteria and reduce inflammation, yet it is hard to dissolve and poorly absorbed by the body, limiting its practical use. In this study, we first collected samples from diseased sows to find the main disease-causing bacteria and tested their sensitivity to antibiotics. We then made tiny particle carriers to wrap curcumin, so curcumin can work better. Our tests showed these particles are safe and can release curcumin efficiently in acidic environments. Both lab and mice experiments proved the particles can kill bacteria, ease uterine tissue damage and reduce inflammation by regulating immune cell behavior. This research provides a new treatment option without antibiotics, which may help cut antibiotic use and slow drug resistance in pig farming. More tests on sows will be carried out before real farm application.

## 1. Introduction

Uterine endometritis is a common postpartum disease in swine, mainly caused by opportunistic pathogens such as *Escherichia coli*, *Staphylococcus* spp., and *Streptococcus* spp. [1,2]. In addition to these commonly reported pathogenic bacteria, *Proteus mirabilis* represents an important opportunistic pathogen contributing to porcine endometritis. It harbors multiple virulence determinants including urease, fimbriae, hemolysin and biofilm-forming capacity, which facilitate bacterial adhesion, intrauterine colonization and subsequent inflammatory injury of endometrial tissue [3]. Field epidemiological investigations have frequently recovered *P. mirabilis* from uterine samples of sows suffering from postpartum or post-artificial-insemination endometritis, which may result in vulvar discharge, return to oestrus and impaired reproductive performance [2]. Currently, conventional antibiotics remain the mainstay of treatment; however, their long-term overuse has led to widespread development of drug-resistant bacteria and marked declines in therapeutic efficacy. Furthermore, antibiotics act solely by targeting pathogenic bacteria, with limited capacity to regulate the excessive inflammatory response triggered by infection. The ensuing cascade of inflammatory mediators further exacerbates tissue damage, perpetuating a vicious “infection-inflammation” cycle [4,5]. This condition not only impairs sow reproductive performance and increases culling rates but also inflicts substantial economic losses on the livestock industry [6]. Consequently, there is a critical clinical demand for new therapeutic approaches that combine strong antibacterial effects with targeted anti-inflammatory control.

Nanomaterials are promising platforms for anti-infective therapy due to their unique physicochemical property [7]. Metal–organic frameworks (MOFs) are attractive as drug carriers because of their large specific surface area, tunable porous structure, and excellent biocompatibility [8,9,10]. Among them, Zeolitic imidazolate framework-8 (ZIF-8) is synthesized by coordinating zinc ions with 2-methylimidazole and is noted for its straightforward synthesis, acid-susceptible degradation, and high drug loading capacity. Notably, ZIF-8 nanoparticles themselves exhibit intrinsic antibacterial activity [11]. ZIF-8′s reduced stability in mildly acidic conditions matches the microenvironment of inflamed tissues, making it suitable for targeted drug delivery to inflamed areas [12].

Curcumin, a turmeric-derived polyphenol, possesses antibacterial, anti-inflammatory, and antioxidant properties [13]. Its antibacterial action involves disrupting bacterial membrane integrity, inhibiting biofilm formation, and interfering with bacterial metabolism [14]. The anti-inflammatory effects result from the inhibition of NF-κB and MAPK pathways, reducing pro-inflammatory cytokine [15,16]. Curcumin’s clinical application is significantly restricted because of its inadequate water solubility, low bioavailability, and chemical instability [17]. Cur@ZIF-8 nanocomplexes, created by encapsulating curcumin in ZIF-8 nanoparticles, improve curcumin stability and targeted delivery, while boosting antibacterial and anti-inflammatory effects together with Zn^2+^ ions. Beyond general advantages for drug delivery, this nanoformulation exhibits favorable properties adapted to intrauterine administration. ZIF-8 nanoparticles may support local drug release within the uterine cavity and help reduce rapid drug clearance by uterine fluid [18]. Furthermore, the nanoscale dimension of Cur@ZIF-8 facilitates superficial penetration into the inflamed endometrial tissue, allowing therapeutic agents to reach infection-associated lesion sites [19]. Although ZIF-8 does not possess strong inherent mucosal-adhesive capacity, the nanoparticulate form can prolong residence time on the endometrial mucosal surface via physical retention, which helps maintain effective local drug concentrations against endometritis-associated pathogens and excessive uterine inflammation [20].

Of note, the in vivo therapeutic efficacy was preliminarily assessed using a mouse endometritis model. Mice are widely adopted pre-clinical animals for anti-endometritis agent screening owing to low cost, easy breeding and well-controlled experimental conditions [21,22]. Nevertheless, inter-species physiological disparities in uterine anatomy, estrous cycle and genital microbiota between mice and sows limit direct translational extrapolation. Therefore, findings obtained from mouse experiments should be interpreted cautiously, and further validation in sow models is required for future practical application [23].

Herein, we report an innovative curcumin-loaded ZIF-8 nanoplatform for treating porcine endometritis, addressing both curcumin’s low bioavailability and concerns over antibiotic overuse. Using a mouse model of porcine endometritis induced by the main clinical pathogen, we further evaluated the in vivo combined therapeutic effects of Cur@ZIF-8, which merges drug carrier and antibacterial capabilities with curcumin’s anti-inflammatory and antibacterial properties. This study offers new insights into nanotherapies for livestock reproductive infectious diseases, supporting the sustainable development of the pig farming.

## 2. Materials and Methods

### 2.1. Experimental Animals and Samples

Forty-eight sows exhibiting clinical signs of endometritis, such as purulent or mucopurulent vaginal discharge, listlessness, decreased feed intake, and irregular estrus, were chosen from a large-scale commercial pig farm. All animals were Landrace × Yorkshire crossbred multiparous sows (parity 2–3), aged 2–3 years, sampled at 3–7 days after farrowing, in lactation period, with purulent vaginal discharge as typical endometritis symptom. Vaginal swabs were collected from each sow under sterile conditions, placed in sterile centrifuge tubes containing 1 mL of sterile normal saline, and transported to laboratory within 2 h after collection under 4 °C refrigeration, and bacterial isolation culture was performed immediately. All clinical sow-derived sample collection procedures were reviewed and approved by the Animal Ethics Committee of Fujian Agriculture and Forestry University (Approval No. PZCASFAFU24007). Mice, aged 6–8 weeks and weighing 18–22 g, were sourced from Fujian Zaiji Biotechnology Co., Ltd. (Fuzhou, China) and kept in a specific pathogen-free (SPF) environment with unlimited access to food and water.

### 2.2. Reagents and Instruments

Curcumin (≥98% purity), ZIF-8, Zn(NO_3_)_2_·6H_2_O, 2-methylimidazole, and dimethyl sulfoxide (DMSO) were sourced from Sigma-Aldrich (St. Louis, MO, USA). Bacterial culture media, MTT reagentand 2,3,5-Triphenyltetrazolium chloride (TTC) was acquired from Beijing Solarbio Science & Technology Co., Ltd. (Beijing, China). Antibiotic susceptibility test discs, including ceftiofur, ampicillin, streptomycin, tetracycline, enrofloxacin, and florfenicol, were obtained from Beckman Biotechnology Co., Ltd. (Shanghai, China). Zoletil^®^ (Virbac, Carros, France) was provided by Fujian Agriculture and Forestry University (Fuzhou, China). HRP-conjugated secondary antibodies and TSA fluorescent staining reagents were obtained from Servicebio (Wuhan, China).

### 2.3. Bacterial Isolation and Culture

Vaginal swab samples were homogenized, and 100 μL of the homogenate was inoculated onto LB agar containing 5% fetal bovine serum (FBS), then incubated at 37 °C for 24 to 48 h. For each sample, colonies were preliminarily screened according to morphological features including colony size, color, margin morphology, surface texture and hemolytic activity. Three representative colonies with distinct morphotypes were picked and subjected to repeated streak-plate purification combined with serial dilution to obtain single-colony isolates. and then were preserved at −80 °C in 25% (*v*/*v*) glycerol for future experiments. For primary bacterial isolation, LB medium supplemented with 5% serum was used to provide additional nutrients and facilitate the recovery of bacteria from clinical sow samples. Mixed bacterial infection was defined when ≥2 distinct bacterial species were identified from isolates recovered from the same vaginal swab sample.

### 2.4. 16S rRNA Sequencing and Identification

Genomic DNA was extracted from purified bacterial strains using the DNA extraction kit (TIANGEN BIOTECH, Beijing, China), following the manufacturer’s standard protocol. The near-full-length 16S rRNA gene was PCR-amplified targeting the V1-V9 hypervariable regions using universal primers: 27F (5′-AGAGTTTGATCMTGGCTCAG-3′) and 1492R (5′-GGTTACCTTGTTACGACTT-3′) [2]. PCR amplification was performed in a 50 μL reaction system under the following conditions: initial denaturation at 95 °C for 5 min; 30 cycles of denaturation at 95 °C for 30 s, annealing at 55 °C for 30 s, and extension at 72 °C for 90 s; followed by a final extension step at 72 °C for 10 min. Purified PCR products were subjected to Sanger sequencing. The obtained sequences were analyzed using the NCBI online BLAST web-server (https://blast.ncbi.nlm.nih.gov/Blast.cgi, accessed on 15 September 2026) against the NCBI GenBank database; bacterial species were assigned based on sequence similarity ≥ 99%.

### 2.5. Antimicrobial Susceptibility Test

The disc diffusion method, in accordance with CLSI guidelines, evaluated the drug sensitivity of isolated pathogenic bacteria. The bacterial suspension was standardized to 0.5 McFarland and uniformly distributed on the Mueller-Hinton (MH) agar. 0.1% TTC was added to MH agar to suppress *P. mirabilis* swarming. Antibiotic susceptibility discs were placed on the medium and incubated at 37 °C for 16 h. The inhibition zone diameter was assessed, and drug sensitivity was categorized as sensitive intermediate, or resistant according to CLSI guidelines (Table S1). *E. coli* ATCC 25922 and *S. aureus* ATCC 25923 were used as CLSI reference quality control strains in every batch of disk diffusion testing.

### 2.6. Preparation and Characterization of Cur@ZIF-8 Nanomaterials

Cur@ZIF-8 nanoparticles were synthesized using the “one-pot method”. 3.25 g of 2-methylimidazole was accurately weighed and dissolved in 100 mL of methanol. 1.47 g of zinc nitrate and 25 mg of curcumin were also accurately weighed and dissolved in 100 mL of methanol. The two solutions were mixed and stirred at a speed of 400 r/min at room temperature for 1 h. An orange-yellow suspension was obtained. The suspension was washed with methanol three times and centrifuged to obtain the yellow Cur@ZIF-8 nanoparticle precipitate. Blank ZIF-8 nanoparticles (without curcumin loading) were also synthesized in-house using the identical one-pot procedure, with the exception that curcumin was omitted from the reaction mixture. Curcumin standard solutions with serial concentrations of 5, 10, 15, 20, 25, and 30 μg/mL were accurately prepared using methanol as the solvent. The ultraviolet-visible (UV-Vis) absorption spectra of the above solutions were scanned within the wavelength range of 200–800 nm, and 420 nm was determined as the characteristic absorption wavelength of curcumin according to the spectral results. A curcumin standard curve was plotted with curcumin concentration as the x-axis and corresponding absorbance value as the y-axis. On this basis, the nanoparticle suspension was centrifuged to separate free curcumin from the nanoparticles. The supernatant containing unencapsulated curcumin was collected, and absorbance was determined at 420 nm. the encapsulation efficiency and drug loading capacity of curcumin were calculated via the indirect method.

The morphology of Cur@ZIF-8 nanomaterials was observed by SEM and TEM (HT7700, Hitachi, High-Tech, Tokyo, Japan). A laser particle size analyzer (ZS90; Malvern Panalytical, Malvern, UK) measured the particle size and zeta potential. Functional groups were identified using Fourier transform infrared spectroscopy (FT-IR) ((Excalibur HE 3100, Varian, Palo Alto, CA, USA). In vitro drug-release performance under different pH environments of Cur@ZIF-8 was evaluated in vitro by dialysis. Briefly, Cur@ZIF-8 suspension was loaded into a dialysis bag (MWCO 3500 Da) immersed in 200 mL release buffer (pH 7.4 mimicking physiological conditions and pH 5.6 simulating the inflammatory microenvironment). The system was incubated at 37 °C under continuous stirring at 100 r/min. At predetermined time-points (0.5, 1, 2, 4, 8, 12, 24, and 48 h), aliquots of release medium were collected for measurement.

### 2.7. Red Blood Cell Hemolysis and MTT Test

Fresh blood from healthy New Zealand white rabbits was collected, anticoagulated with heparin, and washed with normal saline three times to prepare a 2% (*v*/*v*) red blood cell suspension. Cur@ZIF-8 nanomaterials were dissolved in normal saline to prepare solutions at certain concentrations. A 1 mL concentration solution was mixed with 1 mL of a 2% red blood cell suspension and incubated at 37 °C for 1 h. Normal saline was used as the negative control, and 0.1% (*v*/*v*) Triton X-100 as the positive control. Following incubation, the mixture underwent centrifugation at 3000 r/min for 10 min, after which the supernatant’s absorbance was recorded at 545 nm. The hemolysis rate (%) was calculated using the formula: [(A_sample − A_negative incubated with PBS)/(A_positive sample of the Triton-X100 group − A_negative incubated with PBS)] × 100%. A hemolysis rate < 5% was considered non-hemolytic.

The viability of in Raw264.7 macrophages and SV40 mouse endometrial epithelial cells after Cur@ZIF-8 treatment was assessed using the MTT assay. Briefly, cells were seeded into 96-well plates at an appropriate density and cultured overnight. Subsequently, cells were incubated with a series of concentrations of Cur@ZIF-8 for 24 h. MTT reagent was added to each well and incubated for 4 h. The formazan precipitate was dissolved, and absorbance was measured at 490 nm using a microplate reader. Cell viability was calculated relative to the untreated control group, as previously described [24].

### 2.8. Toxicity Test in Mice

Ten BALB/c mice were divided into two groups of five randomly. One group was administered Cur@ZIF-8 at 50 μg/mL, while the other received normal saline. The mice were administered via intrauterine administration once a day for 14 consecutive days. The general condition, body weight and mortality of the mice were observed and recorded daily. On the 14th day, mice were anesthetized with tiletamine-zolazepam (Zoletil) via intramuscular injection prior to retro-orbital blood collection. After blood sampling, the animals were euthanized by cervical dislocation under Zoletil anesthesia. Blood was collected and used for testing blood routine and biochemistry. The mice were then sacrificed, and their major organs (heart, liver, spleen, lung, kidney, brain, ovary, uterus) were collected for histopathological analysis to evaluate the toxicity of Cur@ZIF-8 NPs.

### 2.9. Determination of MIC and Instant Antibacterial Effect

The MIC of Cur@ZIF-8 nanoparticles was assessed using a standard method. Ten different concentrations were employed in the experiments, and the control groups were divided into a enrofloxacin (200 μg/mL) control group and a negative control group, with two technical replicate wells set for each treatment. A 100 μL bacterial suspension was first introduced into each well of a sterile 96-well plate, then Cur@ZIF-8 NPs were added at different concentrations (80 μL). The plates were incubated at 37 °C for 16–18 h, after which 0.625% Resazurin solution (20 μL) was added to the bacterial cells, followed by an additional 4 h incubation at the same temperature [11,25]. The antibacterial effect was evaluated by noting the solution’s color change following the second incubation.

The antibacterial efficacy of Cur@ZIF-8 nanoparticles was evaluated against three bacterial strains. For *P. mirabilis*, 0.1% TTC was supplemented to the agar medium to suppress swarming migration during the colony counting procedure. Each strain was grown aerobically overnight at 37 °C in appropriate media, centrifuged at 6000 r/min, resuspended in fresh sterile medium, and adjusted to 1 × 10^6^ cfu/mL through gradient dilution. Antibacterial assay utilized a sterile 96-well microplate, with each well containing 100 µL of bacterial suspension and 100 µL of Cur@ZIF-8 at concentrations of 800, 400, 200, 100, and 50 µg/mL. After mixing, the final working concentrations of Cur@ZIF-8 were 25, 50, 100, 200, and 400 μg/mL, respectively. And PBS controls were set. After a 2 h incubation at 37 °C, 100 µL from each well was plated on agar and incubated overnight at 37 °C, followed by colony counting and photography. Tests were conducted in triplicate to ensure reliability.

### 2.10. Establishment and Treatment of Mouse Endometritis Model

Forty BALB/c mice were randomly assigned to eight groups: a normal control group, three monomicrobial infection model groups, three corresponding treatment groups for each infection model, and a Cur@ZIF-8 (50 μg/mL) group. Prior to model induction, estrous cycle synchronization was performed in all female BALB/c mice. Estrogen was administered via intraperitoneal injection 3 days before bacterial inoculation to synchronize estrous stages and ensure uniform uterine physiological status for subsequent infection. Except for the normal control group and Cur@ZIF-8 group, mice in the three model groups and three treatment groups received vaginal bacterial inoculation to establish endometritis models. Mice were anesthetized with Zoletil, and each mouse received a vaginal injection of 50 μL bacterial suspension (1 × 10^6^ CFU/mL). Each model group was inoculated with only one bacterial strain: *S. aureus*, *E. coli*, or *P. mirabilis*, respectively. One day after bacterial infection, each treatment group received the designated dose (total administration volume of 50 μL per mouse) via intrauterine administration. The control groups were treated with PBS solution accordingly. Following intrauterine injection of bacterial suspension or drug solution under anesthesia, mice were maintained supine with elevated hips for about 5 min to prevent uterine fluid reflux. Treatments were performed once daily for 3 consecutive days.

### 2.11. Autopsy Observation and Histopathological Section Analysis

On the fifth day, the animals were euthanized under Zoletil anesthesia, and their uteri were collected. Gross uterine lesions including edema, hemorrhage, and hyperemia were observed and documented. The left half of each uterine tissue were processed by fixation in 4% paraformaldehyde, paraffin embedding, 4 μm sectioning, hematoxylin–eosin staining, and light microscopy examination (CX31; Olympus, Tokyo, Japan). Table S2 displays the microscopic observations and scores for endometrial injury, inflammatory cell infiltration, and tissue edema. The total score of the four items is the final score.

### 2.12. Detection of Bacterial Load in Mouse Uterine Tissues

The right half of each uterine tissue was aseptically collected, weighed to obtain 50 mg per sample, and transferred into a 1.5 mL centrifuge tube. After adding 1 mL of sterile PBS, the tissue was homogenized using a sterile homogenizer. The uterine tissue homogenate was serially diluted with sterile PBS. A 100 μL aliquot of each diluted homogenate was evenly spread onto MH agar plates using a sterile spreader, with three replicate plates prepared for each dilution. The agar plates were incubated upside down in a biochemical incubator for 18 h, followed by bacterial colony enumeration. Colonies with atypical morphology inconsistent with the inoculated strain were excluded to avoid counting non-target microorganisms. Plates with 30–300 discrete single colonies were counted according to standard microbiological criteria. The limit of detection (LOD) for this assay using 50 mg uterine tissue suspension was defined as 5 CFU per 50 mg uterine tissue.

### 2.13. Immunofluorescence Staining for Macrophage Polarization and Cytokine Detection in Uterine Tissue

Uterine tissue sections were blocked with 5% BSA. Subsequently, sequential TSA multiplex immunofluorescence staining was performed. Briefly, tissue sections were incubated with the primary antibody against CD86 (Cell Signaling Technology, Danvers, MA, USA, Cat. No. 19589, Clone: E5W6H, 1:400) overnight at 4 °C, followed by HRP-conjugated secondary antibody incubation and TSA-green (iF488-Tyramide, Servicebio, Wuhan, China, Cat. No. G1231) fluorescence deposition to label M1-type macrophages. After complete antibody stripping, the sections were incubated with CD206 primary antibody (Cell Signaling Technology, Danvers, MA, USA, Cat. No. 24595, Clone: E6T5J, 1:600), followed by HRP secondary antibody and TSA-red (iF594-Tyramide, Servicebio, Wuhan, China, Cat. No. G1242) fluorescence labeling for M2-type macrophages. Finally, F4/80 primary antibody (Abcam, Cambridge, UK, Cat. No. ab300421, Clone: EPR26545-166, 1:1000) was incubated, followed by HRP secondary antibody and TSA-yellow (iF555-Tyramide, Servicebio, Wuhan, China, Cat. No. G1233) fluorescence amplification to label total macrophages. All sections were counterstained with DAPI (Servicebio, Wuhan, China, Cat. No. G1012) for nuclear localization. Images (five random non-overlapping fields) were acquired via confocal microscope (FV10-ASM; Olympus Microsystems, Tokyo, Japan) and analyzed for positive cell quantification using ImageJ software (version 1.54p, National Institutes of Health, Bethesda, MD, USA).

Accurate weights of uterine tissue samples were obtained from each group. Tissues were rapidly homogenized on ice using pre-cooled PBS at a 1:9 mass-to-volume ratio to minimize protein degradation. Tissue homogenate was centrifuged at 12,000 r/min for 15 min at 4 °C. The supernatant was carefully collected on ice for cytokine analysis. TNF-α and IL-6 levels in uterine tissue supernatant were quantified using ELISA kits obtained from Beijing Solarbio Science & Technology Co., Ltd. Cytokine concentrations detected by ELISA were normalized to wet uterine tissue weight and expressed as pg/g tissue.

### 2.14. Statistical Analysis

Sample size *n* = 5 per group was selected for this exploratory proof-of-concept study. This study was not statistically powered to detect subtle low-magnitude toxic changes. SPSS 26.0 software (IBM Corp., Armonk, NY, USA) was used for all data analyses. Multi-group comparisons were conducted using one-way ANOVA followed by Duncan’s multiple comparison post hoc tests. Experiments were conducted at least three times, with statistical significance defined as *p* < 0.05. All quantitative data were expressed as mean ± standard deviation (Mean ± SD). Before statistical comparison, the Shapiro–Wilk test was used to verify data normality, and Levene’s test was used to examine homogeneity of variance. Parametric analyses were applied if both assumptions were met.

## 3. Results

### 3.1. Identification and Drug Sensitivity of Pathogenic Bacteria

**Figure 1:**
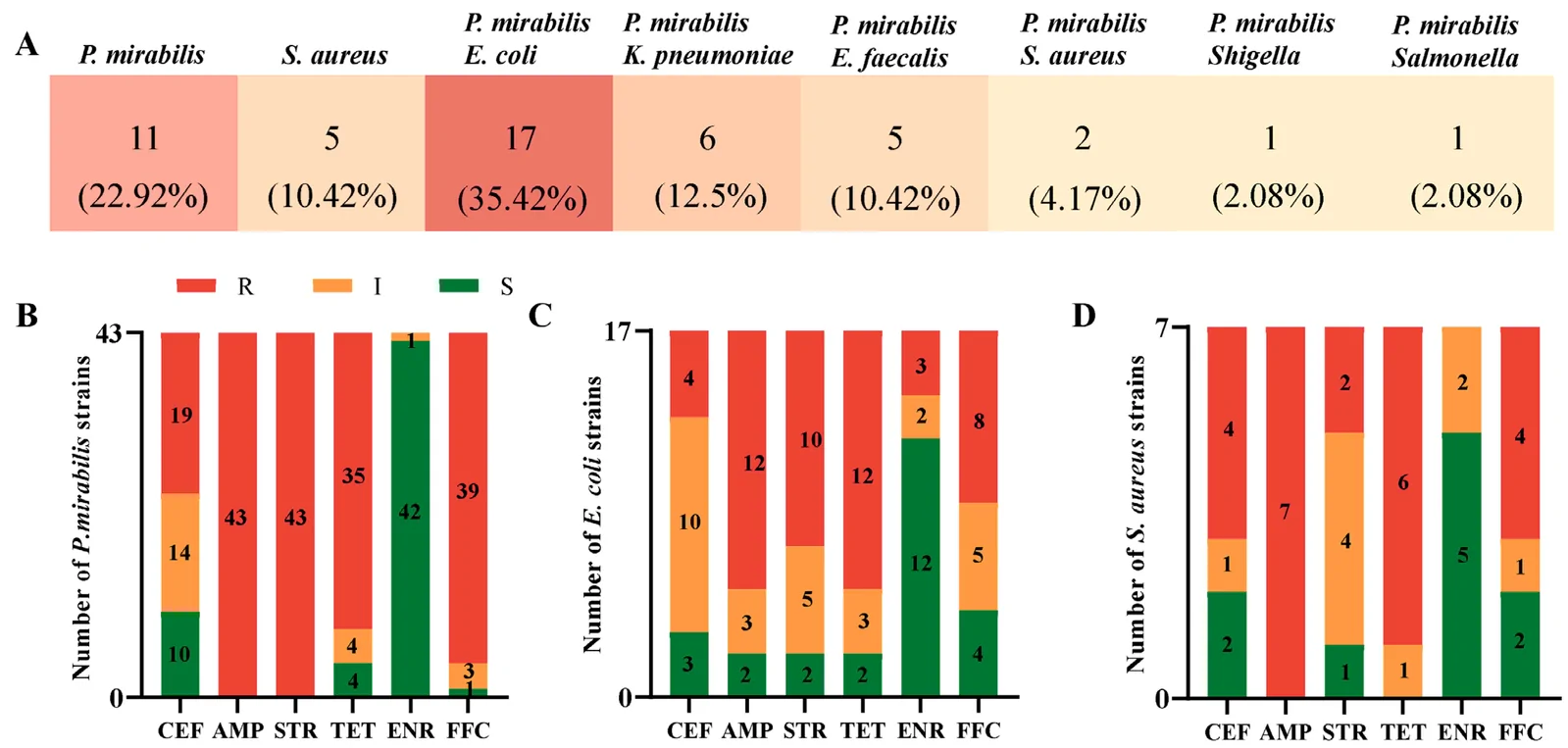
Results of bacterial isolation and identification (**A**) and antibiotic resistance analysis (**B**–**D**) of three dominant pathogenic strains.

Analysis of 48 vaginal swab samples from sows with endometritis using 16S rRNA sequencing identified eight bacterial infections (Figure 1A and Table S3). *Proteus mirabilis* was the predominant pathogen, present in 89.6% (43/48) of cases. This included 11 cases of single infection (22.9%) and mixed infections with *Staphylococcus aureus* (2 cases, 4.17%), *Escherichia coli* (17 cases, 35.4%), *Klebsiella pneumoniae* (6 cases, 12.5%), *Enterococcus faecalis* (5 cases, 10.4%), *Shigella* (1 case, 2.1%), and *Salmonella* (1 case, 2.1%). Additionally, there were 5 cases of single infection with *Staphylococcus aureus* (10.4%).

Antimicrobial susceptibility test results showed that most of the isolated pathogenic bacteria were resistant to commonly used antibiotics (Supplementary Tables S1 and S4). Antimicrobial susceptibility testing of 43 *Proteus mirabilis* isolates revealed extremely high resistance rates. Notably, 100% resistance was observed against both ampicillin (AMP) and streptomycin (STR). Among other agents, florfenicol (FFC) displayed the broadest resistance (90.7%, 39/43), followed by tetracycline (TET, 81.4%, 35/43) and cephalosporin (CEF, 44.2%, 19/43). In stark contrast, enrofloxacin (ENR) exhibited the highest activity, with 42 isolates (97.7%) being susceptible (Figure 1B). Results for 17 *E. coli* isolates indicated severe resistance to AMP and TET, with identical resistance rates of 70.6% (12/17). Moderate resistance was detected against florfenicol (FFC, 47.1%, 8/17) and streptomycin (STR, 58.8%, 10/17). Cefotaxime (CEF) showed a relatively lower resistance rate of 23.5% (4/17). ENR remained the most effective agent, rendering 70.6% (12/17) of the isolates susceptible (Figure 1C). Among the 7 *S. aureus* isolates, high resistance rates were prevalent against AMP and TET, with resistance rates of 100% (7/7) and 85.7% (6/7), respectively. CEF and FFC resistance were moderate at 57.1% (4/7) for both agents. Conversely, STR and ENR demonstrated relatively better efficacy, with susceptibility rates of 14.3% (1/7) and 71.4% (5/7), respectively (Figure 1D).

Collectively, these results highlighted distinct and severe resistance patterns across the three pathogens. *Proteus mirabilis* exhibited universal resistance (100%) to AMP and STR. *E. coli* was most resistant to TET, while *Staphylococcus aureus* also displayed high resistance to AMP and TET. These findings underscore the urgent need for non-antibiotic antibacterial nanoformulations to combat these resistant pathogens.

### 3.2. Characterization of Cur@ZIF-8 Nanomaterials

**Figure 2:**
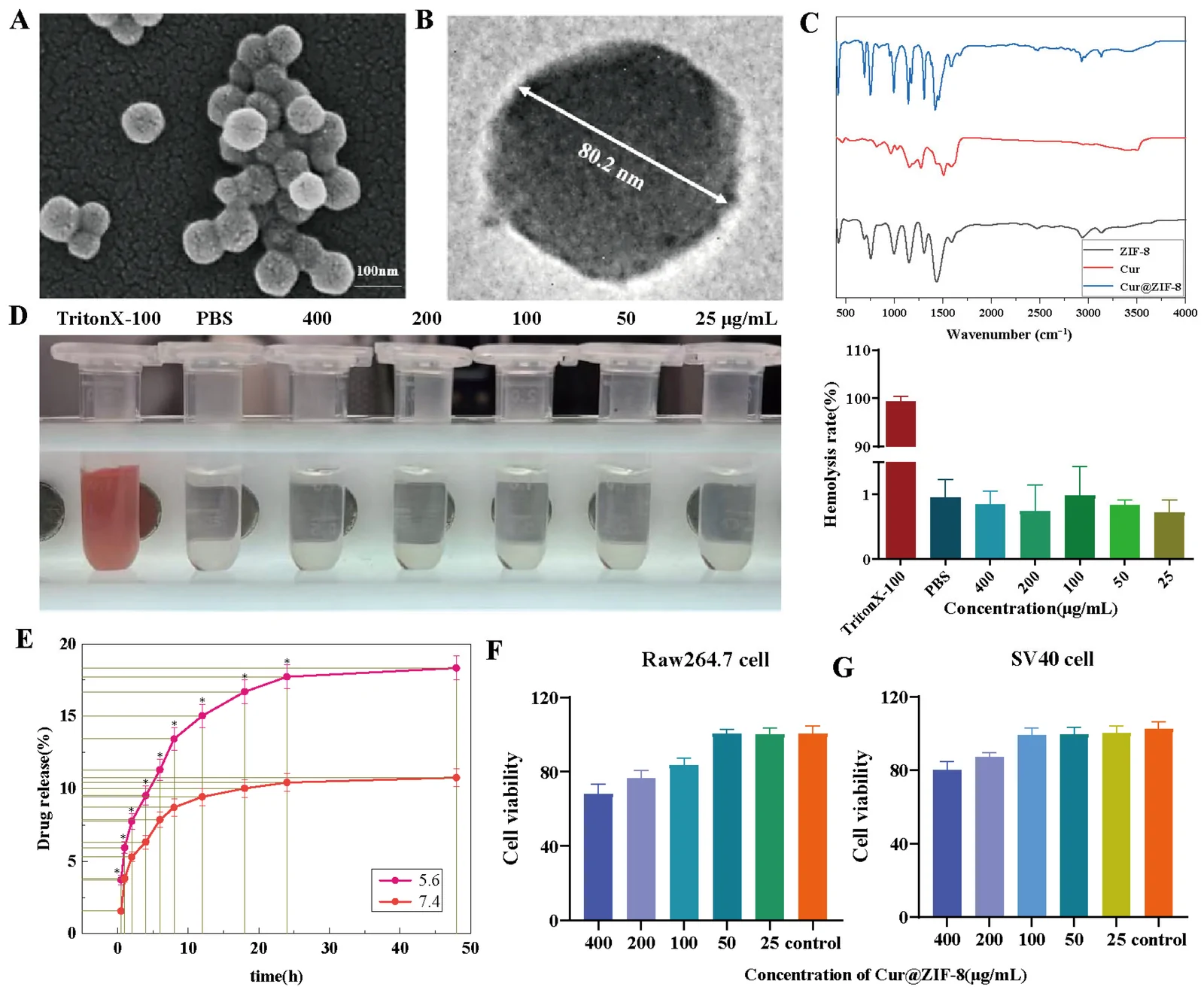
Physicochemical characterization and evaluation of in vitro toxicity of Cur@ZIF-8 NPs. (**A**) Scanning electron microscopy image of Cur@ZIF-8 NPs; (**B**) Transmission electron microscopy image of Cur@ZIF-8; (**C**) FTIR spectra of Cur@ZIF-8; (**D**) Hemolysis rate analysis of Cur@ZIF-8 nanoparticles; (**E**) In vitro curcumin drug release profiles of Cur@ZIF-8 nanoparticles at different pH values, *, *p* < 0.05; (**F**,**G**) Cytotoxicity results of Cur@ZIF-8 nanoparticles.

SEM analysis revealed that Cur@ZIF-8 nanomaterials were uniformly sized, well-dispersed tetrahedral particles (Figure 2A). The particle size was mainly distributed with an average particle size of 80.2 ± 8.5 nm in TEM image (Figure 2B). The average hydrodynamic particle size is 106.2 nm, and the nanomaterials exhibited a zeta potential of 33.9 mV (Figure S1), suggesting good stability and resistance to aggregation. Taking the concentration of cur as the abscissa and the absorbance value at 420 nm as the ordinate for linear analysis, a concentration-absorbance standard curve was plotted: y = 0.0179 x − 0.0191 (R^2^ = 0.9921) (Figure S2). The encapsulation efficiency of the drug was calculated to be 86.4% and the drug loading rate was 10.8% by an indirect method. The in vitro drug release test showed that Cur@ZIF-8 displayed distinct pH-responsive release characteristics. Within 48 h, the drug release rate was 10.7% in a pH 7.4 buffer solution, compared to 18.3% in a pH 5.6 buffer solution. Significant differences in cumulative drug release were observed between the two conditions at all time points (Figure 2E). This indicates that Cur@ZIF-8 maintains relatively high stability under physiological neutral conditions, and exhibits moderately enhanced curcumin release under acidic inflammatory conditions, albeit with restricted overall cumulative release within 48 h.

### 3.3. Biocompatibility and Safety of Cur@ZIF-8 Nanomaterials

The hemolysis test revealed that Cur@ZIF-8 nanomaterials maintained a hemolysis rate below 5% at all concentrations, indicating excellent hemocompatibility (Figure 2D). The in vitro biocompatibility of Cur@ZIF-8 was evaluated by MTT assay. Figure 2F,G illustrates a dose-dependent reduction in cell viability with higher concentrations of Cur@ZIF-8. When the concentration was no more than 50 μg/mL, the viability of both cell lines was comparable to that of the control group, indicating no obvious cytotoxicity. At a concentration of 400 μg/mL, SV40 cell viability exceeded 80%, indicating endometrial epithelial cells’ high tolerance to Cur@ZIF-8.These results demonstrate that Cur@ZIF-8 exhibits favorable in vitro biocompatibility within the potential safe dose range (50 μg/mL) for subsequent intrauterine administration in mice, without significant cytotoxic risks.

**Figure 3:**
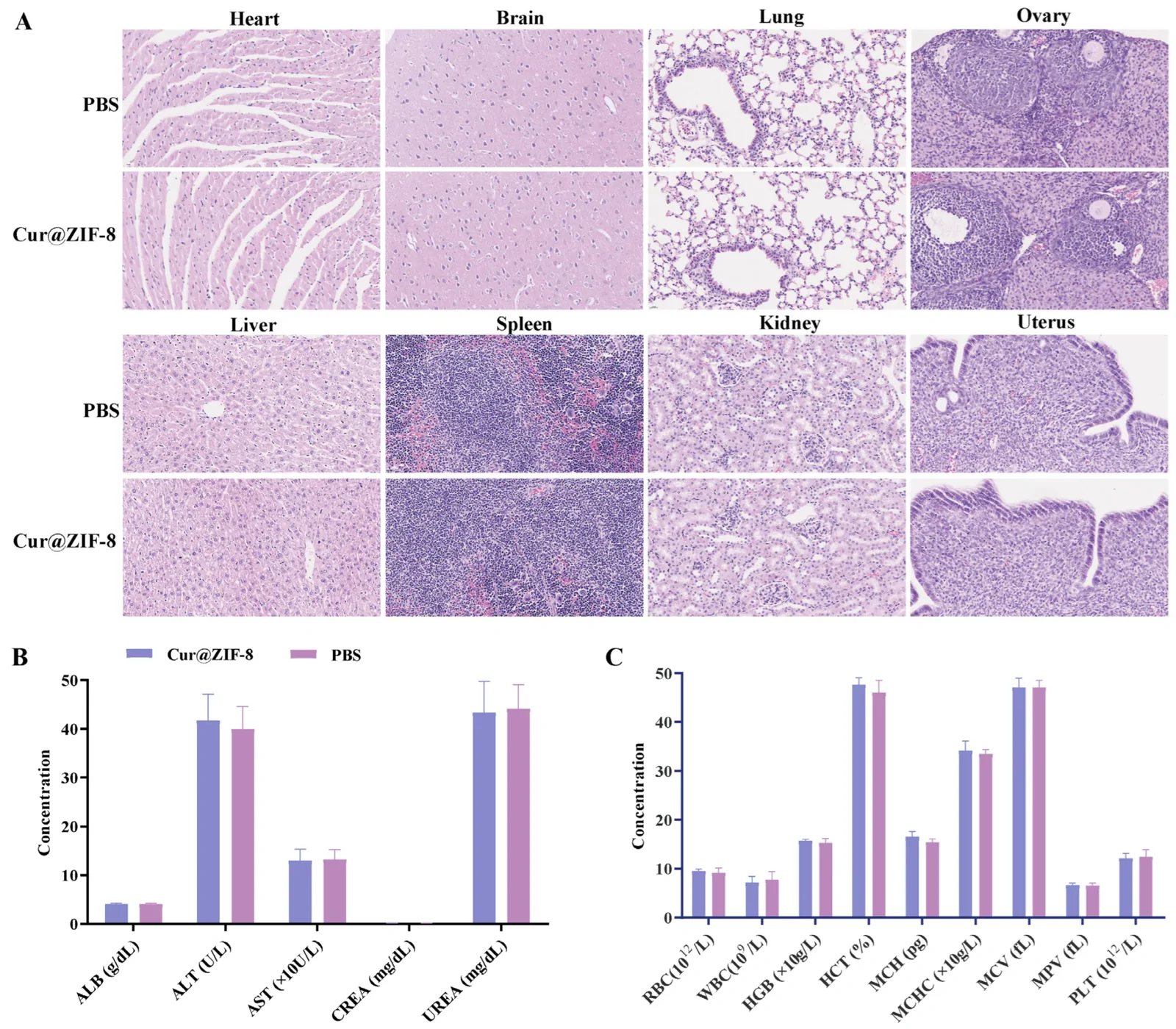
Biocompatibility of Cur@ZIF-8 NPs in mice. (**A**) Histopathological sections of major organs of mice (HE staining, ×200); (**B**) Blood biochemical analysis of mice treated with Cur@ZIF-8 nanoparticles; (**C**) Blood routine analysis of mice treated with Cur@ZIF-8 nanoparticles.

Mouse toxicity test results showed that all mice in each group had normal general condition, no obvious abnormal behavior, and no mortality during the 14-day observation period. Histopathological analysis showed no damage or toxicity to the normal structure of major organs in treated mice. Histopathological analysis showed no notable damage, edema, or inflammatory cell across all mice of treatment group, consistent with the PBS group (Figure 3A). Hematological and biochemical blood analyses indicated no significant differences between the treatment and control group (Figure 3B,C). These in vivo findings suggest Cur@ZIF-8 nanoparticles exhibit high biocompatibility and are safe and reliable for drug delivery in clinical applications within the mouse uterus.

### 3.4. In Vitro Antibacterial Effect of Cur@ZIF-8 NPs

**Figure 4:**
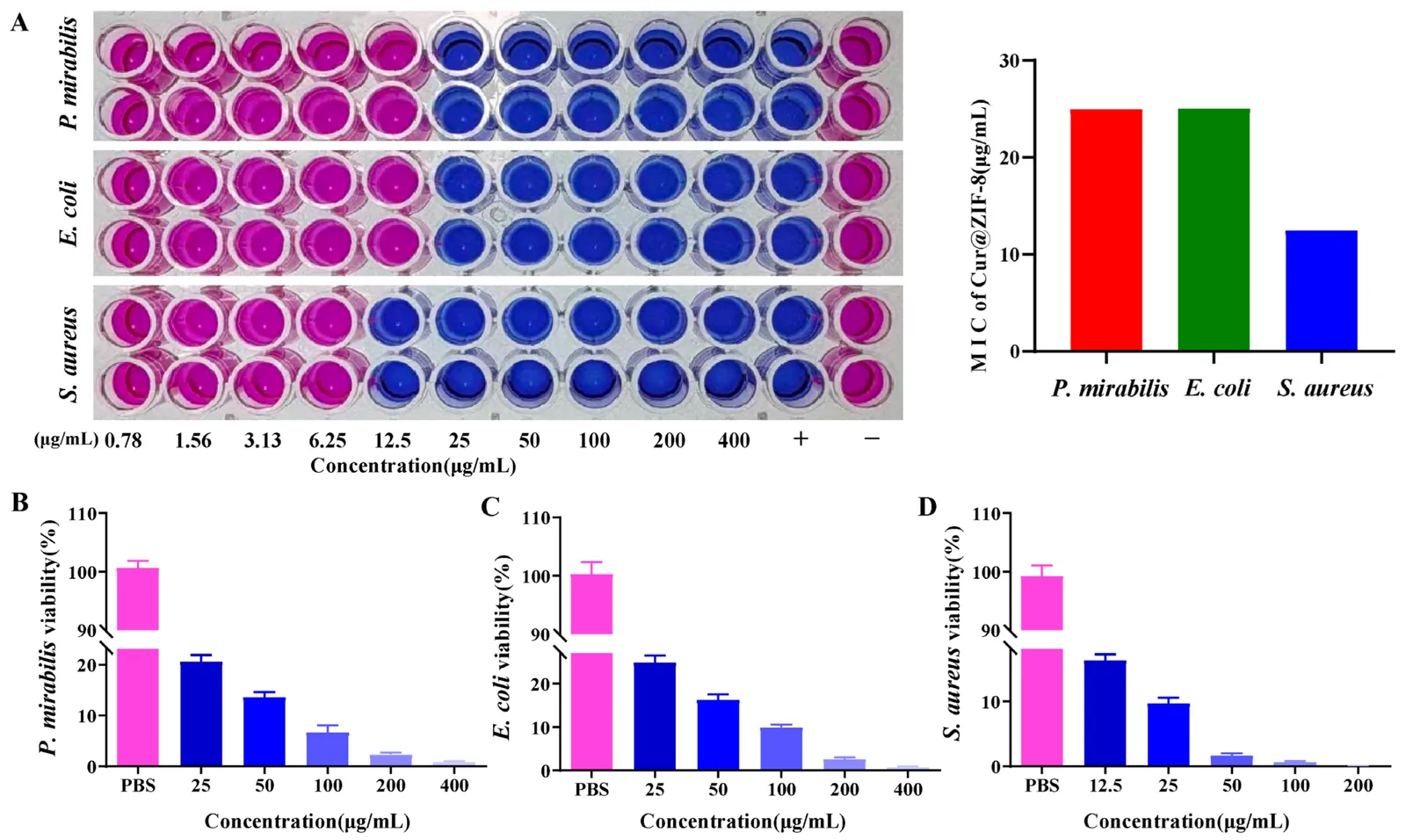
Evaluation of the in vitro antibacterial properties of Cur@ZIF-8 NPs. (**A**) The minimum inhibitory concentration of Cur@ZIF-8 nanoparticles was assessed against three primary pathogenic bacteria responsible for porcine endometritis; (**B**–**D**) The relative viability of these dominant bacteria was evaluated following treatment with Cur@ZIF-8 nanoparticles.

The antibacterial effectiveness of Cur@ZIF-8 against *Proteus mirabilis*, *E. coli*, and *Staphylococcus aureus* was evaluated in vitro using the resazurin microtiter assay and plate counting method. Resazurin, a redox indicator, changes from blue (non-fluorescent, oxidized state) to pink (fluorescent, reduced state) in the presence of metabolically active bacteria. As shown in Figure 4A, the color of the bacterial suspension wells remained pink at low Cur@ZIF-8 concentrations, indicating sustained bacterial viability. In contrast, wells containing high Cur@ZIF-8 concentrations exhibited a blue color, reflecting suppressed bacterial metabolic activity and bacterial growth inhibition. The MIC values were 12.5 μg/mL for *Proteus mirabilis* and 25 μg/mL for both *E. coli* and *Staphylococcus aureus*, indicating the minimum concentrations needed to inhibit visible bacterial growth.

The bactericidal effect of Cur@ZIF-8 was quantified by assessing the relative bacterial viability after 2 h of incubation with varying concentrations of Cur@ZIF-8. Aligned with the resazurin assay findings, Cur@ZIF-8 demonstrated a dose-dependent antibacterial activity against all three tested strains. At MIC concentrations, the viability of *Proteus mirabilis*, *E. coli*, and *Staphylococcus aureus* significantly decreased to approximately 20%, 10%, and 25%, respectively, relative to the PBS control group. At 50 μg/mL, Cur@ZIF-8 nanoparticles demonstrated strong antibacterial activity, reducing bacterial viability to less than 1% across all three strains (Figure 4B–D).

### 3.5. Therapeutic Effect of Cur@ZIF-8 Nanomaterials on Mouse Endometritis

#### 3.5.1. Autopsy Observation Results

**Figure 5:**
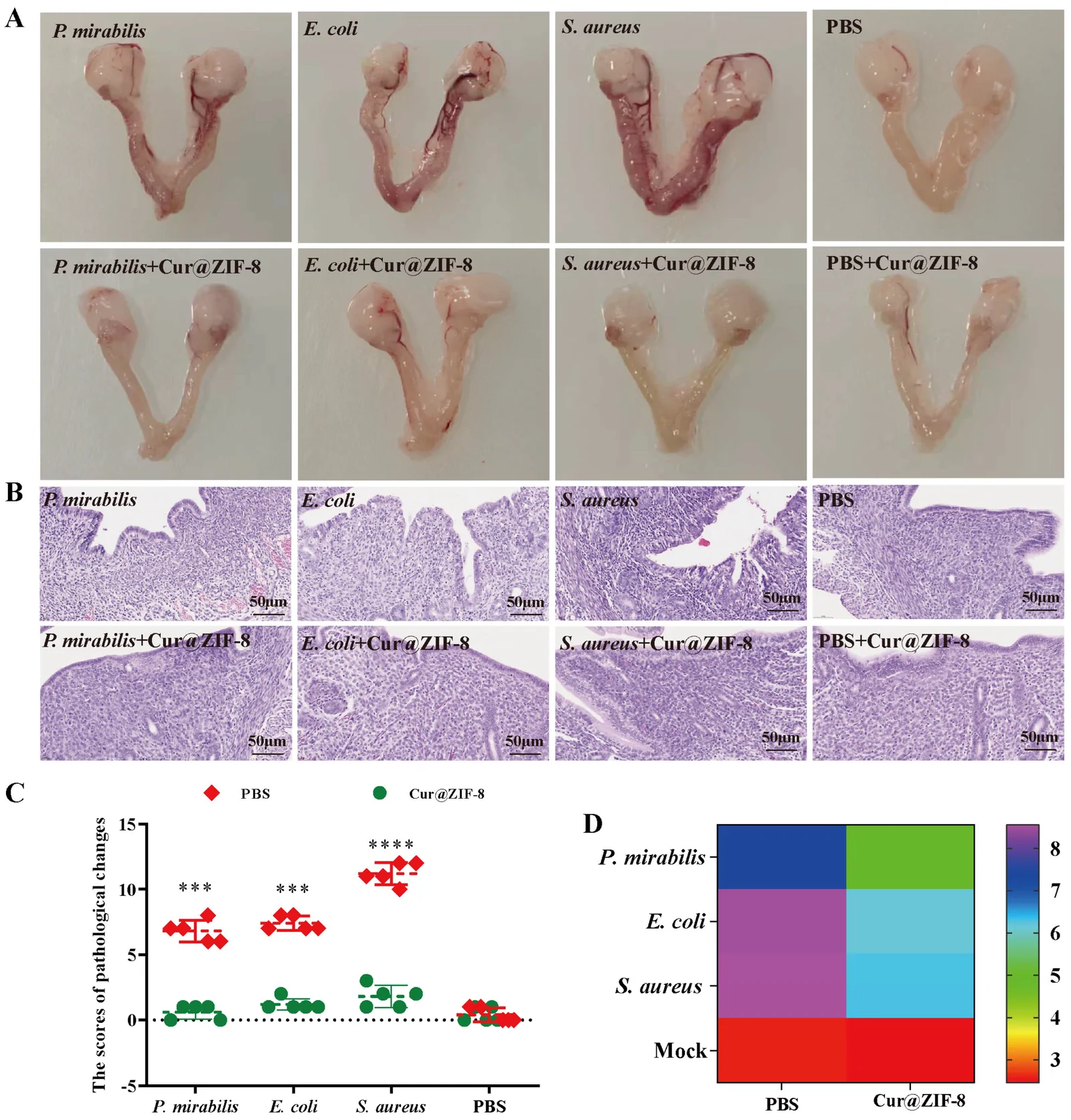
Evaluation of the therapeutic effect on mice infected with three dominant pathogenic bacteria of porcine endometritis. (**A**) Gross examination, (**B**,**C**) H&E staining with histopathological scoring, and (**D**) bacterial load assessment of uterine tissue post-treatment with Cur@ZIF-8 nanoparticles (log_10_(CFU)), ***, *p* < 0.001; ****, *p* < 0.0001.

Daily body weight of mice was monitored throughout the in vivo experiment. No significant inter-group differences in body weight were observed across all experimental groups during the 5-day observation period, and no obvious treatment-related weight loss occurred. The complete body-weight data are provided in Supplementary Figure S3. Autopsy findings revealed that the uterus in the normal control group was standard-sized, pale red, and free from edema, hemorrhage, or congestion. The uteri in the three model groups were significantly swollen, dark red in color, and had obvious hemorrhage, congestion and edema, with the severity being *Staphylococcus aureus* group > *Escherichia coli* group > *Proteus mirabilis* group (Figure 5A).

The uterus in the Cur@ZIF-8 group was basically normal, without obvious edema, hemorrhage or congestion. HE staining indicated that the normal control group’s endometrium was structurally intact, with orderly epithelial cell arrangement and no inflammatory cell infiltration (Figure 5B,C). The endometrium of the *Staphylococcus aureus* group was severely damaged, with a large amount of neutrophil infiltration, severe edema and necrosis of epithelial cells. The *Escherichia coli* group’s endometrium exhibited mild to moderate damage, characterized by slight inflammatory cell infiltration and mild edema. The *Proteus mirabilis* group’s endometrium exhibited mild damage, minimal inflammatory cell infiltration, and lacked significant edema. The three Cur@ZIF-8 treatment group exhibited a structurally intact endometrium, characterized by an orderly arrangement of epithelial cells and minimal inflammatory cell infiltration, closely resembling the normal control group. Cur@ZIF-8 group exhibited a significantly lower histopathological score compared to the three model groups, with no significant difference from the control group. The findings indicate that Cur@ZIF-8 effectively treats mouse endometritis induced by prevalent clinical strains.

#### 3.5.2. Analysis of Bacterial Load in Mouse Uterine Tissues

Figure 5D presents a heatmap analysis of bacterial loads in uterine tissues of endometritis-afflicted mice treated with Cur@ZIF-8, post-infection by *Proteus mirabilis*, *E. coli*, and *Staphylococcus aureus*. The uterine tissues exhibited extremely high bacterial loads across all three infection models. The highest load was observed in the *Staphylococcus aureus*-infected group, followed by the *E. coli*-infected group and the *Proteus mirabilis*-infected group. In stark contrast, Cur@ZIF-8 treatment significantly reduced the uterine bacterial burden in all infection models. Notably, the bacterial loads in both *E. coli*- and *Proteus mirabilis*-infected groups were reduced to near the detection limit, while the load in the *Staphylococcus aureus*-infected group also decreased substantially. These results demonstrate that Cur@ZIF-8 exhibits excellent in vivo antibacterial activity against all three tested pathogens, effectively eliminating bacteria from the uterine tissue, with particularly potent efficacy against the Gram-negative strains *E. coli* and *Proteus mirabilis*.

#### 3.5.3. Results of Macrophage Polarization and Cytokines

**Figure 6:**
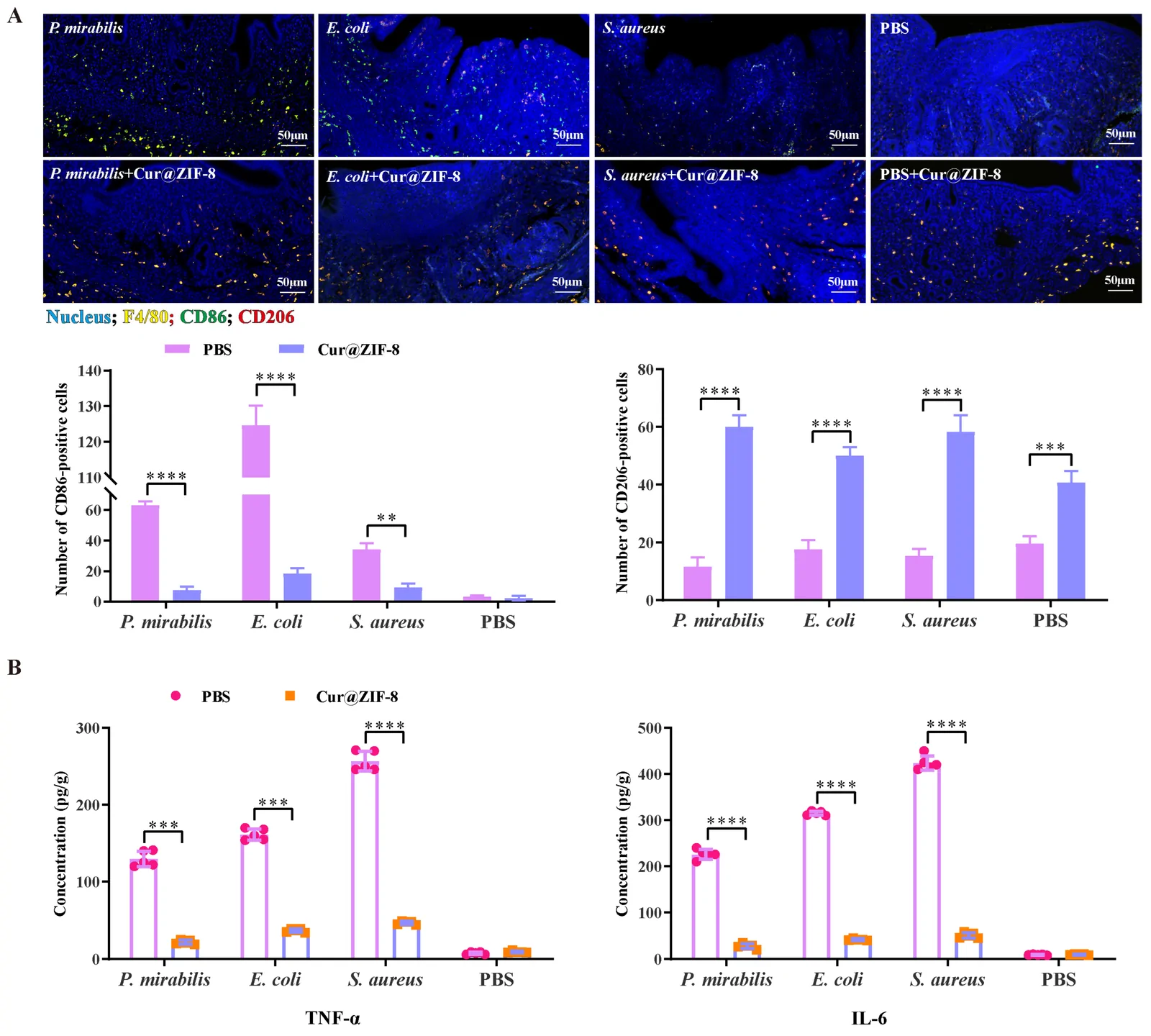
Detection results of macrophage polarization and cytokines in uterine tissue after Cur@ZIF-8 NPs treatment. (**A**) Results of macrophage polarization in uterine tissue after Cur@ZIF-8 NPs treatment; (**B**) Changes in cytokine levels in uterine tissue after Cur@ZIF-8 NPs treatment. **, *p* < 0.01, ***, *p* < 0.001, ****, *p* < 0.0001.

To examine the condition of uterine macrophages, immunofluorescence staining and quantitative analysis were conducted for the M1(CD86) and M2 (CD206) marker proteins (Figure 6A). Cur@ZIF-8 treatment markedly reduced CD86-positive macrophages in all three infection models (*Proteus mirabilis*, *E. coli*, and *Staphylococcus aureus*) compared to the PBS control group, showing significant differences. The blank PBS group exhibited only negligible numbers of CD86-positive cells. Cur@ZIF-8 treatment markedly elevated the count of CD206-positive macrophages across all infection models. Cur@ZIF-8 treatment notably elevated CD206-positive cell counts across *Proteus mirabilis*, *E. coli*, *Staphylococcus aureus* models, and the PBS control group. The results demonstrate that Cur@ZIF-8 treatment effectively inhibits M1 macrophage polarization while promoting M2 polarization, indicating its potential to modulate the inflammatory microenvironment and facilitate inflammation resolution.

We evaluated the in vivo anti-inflammatory effects of Cur@ZIF-8 by measuring pro-inflammatory cytokines TNF-α and IL-6 in mouse tissues following infection with *Proteus mirabilis*, *E. coli*, or *Staphylococcus aureus*, comparing results with and without Cur@ZIF-8 treatment. Figure 6B demonstrates that infection with any of the three bacterial strains induced a robust inflammatory response, evidenced by significantly elevated TNF-α and IL-6 levels in the untreated (PBS) group compared to the uninfected PBS control. Notably, treatment with Cur@ZIF-8 drastically reduced the production of both cytokines across all infection models.

Cur@ZIF-8 treatment significantly lowered TNF-α levels in mice infected with *Proteus mirabilis*, *E. coli*, and *Staphylococcus aureus* compared to those treated with PBS. A similar trend was observed for IL-6, where Cur@ZIF-8 treatment led to a profound reduction in cytokine levels, dropping from over 200–450 pg/g in the infected PBS groups to roughly 40–60 pg/g in the Cur@ZIF-8 groups. These results demonstrate that Cur@ZIF-8 effectively suppresses excessive pro-inflammatory cytokine release in vivo, mitigating the inflammatory response triggered by bacterial infection.

## 4. Discussion

Porcine endometritis is a common reproductive disease in swine production, linked to factors such as pathogenic infections, immune status, and feeding management. The core etiology is the invasion of pathogenic bacteria into the uterine mucosa, which triggers acute or chronic inflammatory responses [1,26]. This disease is predominantly caused by bacterial infection. In this study, pathogenic bacteria were isolated and identified from 48 pigs with endometritis via 16S rRNA sequencing. The results revealed that *Proteus mirabilis* was the dominant strain, followed by *Escherichia coli* and *Staphylococcus aureus*. Previously reported common pathogens mainly include *Escherichia coli*, *Staphylococcus*, *Streptococcus*, *Klebsiella*, and *Pseudomonas aeruginosa* [1,27,28]. This discrepancy may be attributed to the feeding environment, feeding management practices, and antibiotic use in pig farms. Antimicrobial susceptibility test results showed that most of the isolated pathogenic bacteria were resistant to common antibiotics, which is consistent with the current status of antibiotic resistance in the livestock industry [28,29]. Traditional antibiotic treatments are not highly effective against porcine endometritis and can disrupt the microecological balance in pigs, posing public health risks due to drug residues entering the food chain. It should be noted that *P. mirabilis* has intrinsic resistance to several antimicrobial agents including colistin and nitrofurantoin, which represents species-encoded innate resistance [30,31]. In contrast, resistance to β-lactam antibiotics observed in this study is classified as acquired resistance, commonly mediated by horizontally transferred resistance genes such as β-lactamase genes [31]. We differentiated these two categories of resistance phenotypes when interpreting the antimicrobial resistance profiles of isolates. Consequently, it is crucial to create innovative non-antibiotic treatments that are safe, effective, and devoid of antibiotic resistance concerns.

Curcumin demonstrates significant antibacterial and anti-inflammatory properties. However, its clinical use is restricted due to limited water solubility and bioavailability [32,33,34]. After nanocrystallization of curcumin, its antibacterial and anti-inflammatory efficacy has significantly improved. Experimental data indicate that zinc-doped curcumin carbon dots, developed by Professor Li Bing’s team, significantly enhance the regulatory effect on the VEGF signaling pathway and promote healing of infected wounds through the combination of zinc ions and curcumin [35]. Recently, ZIF-8 nanoparticles have been extensively utilized as carriers for various drugs, including antibiotics, natural active components, and chemotherapeutic agents, to improve solubility, targeting ability, and bioavailability. ZIF-8 nanoparticles compromise bacterial cell membrane integrity, causing intracellular leakage, arginine biosynthesis inhibition, increased reactive oxygen species accumulation, and tricarboxylic acid (TCA) cycle disruption, ultimately leading to bacterial death [11]. In this study, Cur@ZIF-8 nanomaterials were prepared and characterization results demonstrated that these nanomaterials had uniform particle size, good dispersion, and pH-responsive drug release properties. The drug loading and encapsulation efficiencies surpassed those of other curcumin nanodelivery systems [36,37]. In vitro assays revealed that Cur@ZIF-8 maintains relatively good stability under normal physiological conditions, while curcumin liberation is elevated under acidic inflammatory microenvironments. It should be noted that approximately 81.7% of curcumin remained encapsulated within nanoparticles after 48 h of in vitro release test. This incomplete release may be partially attributed to strong hydrophobic interaction between curcumin molecules and the porous ZIF-8 framework. The substantial retained drug fraction inside nanoparticles may serve as an internal drug reservoir, which could sustain local drug supply over an extended time window within the uterine cavity. Nevertheless, incomplete drug liberation also implies that only a portion of loaded curcumin participates in immediate therapeutic actions, which may constrain instantaneous pharmacological potency. This feature should be considered when interpreting in vivo efficacy duration, and further optimization is needed to tune release kinetics for clinical translation. ** Such differential release behavior helps elevate curcumin levels at lesion sites and minimizes adverse effects on healthy tissues.

Biocompatibility and safety are crucial prerequisites for the clinical application of nanomaterials [38]. The study’s red blood cell hemolysis tests revealed that Cur@ZIF-8 maintained a hemolysis rate under 5% at all concentrations, indicating superior blood compatibility. Mouse toxicity tests confirmed that Cur@ZIF-8 exhibited no significant toxicity or organ damage in mice, aligning with prior findings on ZIF-8’s safety as a drug carrier [12,39]. It is worth noting that ZIF-8 undergoes gradual degradation in physiological environments, releasing zinc ions and 2-methylimidazole as main degradation products. Published studies have demonstrated that appropriately dosed Zn^2+^ participates in normal cellular metabolism and exhibits good biocompatibility, while high local or long-term cumulative exposure to degradation products may trigger inflammatory responses or cytotoxicity [40,41]. In the present study, acute safety tests confirmed the satisfactory short-term biocompatibility of Cur@ZIF-8. Of note, larger animal cohorts will be required in future dedicated safety studies to assess minor toxic alterations. The results demonstrate that Cur@ZIF-8 nanomaterials possess excellent biocompatibility and safety, rendering them appropriate for in vivo therapeutic use. Nevertheless, these therapeutic observations are derived from single-pathogen mouse models; caution should be exercised when extrapolating such findings to natural polymicrobial porcine endometritis under farm settings. The mouse model of endometritis, induced through intratureine injection of *Staphylococcus aureus*, *E. coli*, and *Proteus mirabilis*, successfully mirrors the clinical pathological features of porcine endometritis, including uterine edema, hemorrhage, congestion, inflammatory cell infiltration, and tissue necrosis. Efficacy evaluation results showed that Cur@ZIF-8 nanomaterials could significantly improve the uterine pathological state, reduce the degree of endometrial damage and inflammatory cell infiltration, and greatly decrease the bacterial load in the infected groups. Cur@ZIF-8 enables targeted curcumin release in inflammatory settings, boosting the combined antibacterial and anti-inflammatory properties of curcumin and ZIF-8. Previous studies in our laboratory have also demonstrated that ZIF-8 alone exhibits excellent therapeutic effects against *E. coli*, *Staphylococcus aureus*, and even MRSA in a mouse skin infection model [11]. Notably, Cur@ZIF-8 exerts broad-spectrum antibacterial effects against both Gram-positive and Gram-negative bacteria via distinct mechanisms [42,43]. For Gram-negative strains, positively charged Cur@ZIF-8 adheres to the bacterial outer membrane, disrupting membrane integrity, increasing permeability, and inducing excessive ROS accumulation. For Gram-positive bacteria without an outer membrane, Cur@ZIF-8 mainly damages the peptidoglycan layer, disturbs intracellular metabolism, and inhibits arginine synthesis. Such differential bactericidal behaviors endow Cur@ZIF-8 with efficient broad-spectrum antibacterial activity against endometritis-associated pathogens.

Macrophage polarization is crucial in modulating inflammatory responses. M1 macrophages secrete pro-inflammatory factors that exacerbate inflammation, while M2 macrophages release anti-inflammatory factors that aid in resolving inflammation and facilitating tissue repair [44]. Immunofluorescence staining results showed that Cur@ZIF-8 could reduce M1-type macrophage polarization and increase M2-type macrophage polarization in the three infected mouse groups, regulating the balance of macrophage polarization to exert anti-inflammatory effects. Administration of ZIF-8 or curcumin-loaded ZIF-8 (Cur@ZIF-8) significantly decreased TNF-α and IL-6 levels in uterine tissue compared to the untreated model group. Previous research indicates that curcumin modulates macrophage polarization to mitigate inflammatory bowel disease in mice, though the precise molecular mechanism remains to be explored [45]. ZIF-8 nanoparticles facilitate the release of Zn^2+^ ions, which promote the conversion of M1-type macrophages to M2-type macrophages, thereby supporting tissue repair, consistent with the findings of this study [46].

The study is subject to several limitations. First, clinical isolates were recovered from vaginal swabs of sows with suspected endometritis under farm conditions. Vaginal swabs cannot completely rule out contamination by commensal flora from the lower genital tract. Although we selected sows with typical clinical symptoms and used standardized sampling procedures to reduce contamination, intrauterine sampling would be ideal to identify true endometrial pathogens. In addition, primary isolation using LB medium supplemented with 5% serum may fail to recover highly fastidious bacteria requiring specialized media. Second, all bacterial isolation was performed aerobically, so obligate anaerobic pathogens associated with porcine endometritis were not captured, which should be considered when interpreting our pathogen distribution data. Third, therapeutic efficacy and the detailed antibacterial mechanism of Cur@ZIF-8 against our clinical isolates and the molecular pathway governing macrophage polarization was verified in a mouse model; further validation in pigs is necessary. Fourth, only the PBS control group was set for in vivo therapeutic evaluation. Free curcumin, blank unloaded ZIF-8, and antibiotic positive controls were absent, preventing us from distinguishing the individual effects of curcumin and ZIF-8 or comparing Cur@ZIF-8 with standard antibiotics. The present study aimed to preliminarily evaluate the overall protective effect of Cur@ZIF-8, and follow-up mechanistic research will incorporate these key control groups. Fifth, independent single-pathogen mouse models were used for in vivo tests, whereas natural porcine endometritis is usually polymicrobial. Single-strain models avoid interbacterial interference and allow precise assessment of Cur@ZIF-8 against each pathogen, yet they cannot replicate the complex polymicrobial microenvironment of clinical cases. Future studies will establish polymicrobial co-infection models to better simulate natural disease.

## 5. Conclusions

This study utilized 16S rRNA sequencing to identify the bacterial spectrum associated with porcine endometritis, revealing that *Proteus mirabilis*, *Staphylococcus aureus*, and *Escherichia coli* were the predominant pathogenic strains, most of which exhibited resistance to conventional antibiotics. The synthesized Cur@ZIF-8 nanomaterials presented favorable physicochemical characteristics and excellent biocompatibility. In murine single-pathogen endometritis models, Cur@ZIF-8 achieved powerful in vivo antibacterial efficacy, reducing uterine bacterial loads to near the detection limit in *E. coli* and *P. mirabilis* infection models and substantially decreasing bacterial colonization in *S. aureus*-infected uterine tissues. In terms of anti-inflammatory performance, Cur@ZIF-8 treatment drastically suppressed pro-inflammatory cytokine release, decreasing uterine IL-6 levels from 300 to 450 pg/g in the untreated model group to below 50 pg/g. Meanwhile, Cur@ZIF-8 effectively inhibited M1 macrophage polarization and promoted M2 macrophage polarization, thereby remodeling the inflammatory microenvironment and alleviating endometrial tissue injury.

Notably, all therapeutic evaluations in this study were performed in mouse models, and the current findings should be regarded as preliminary pre-clinical results. Due to inherent interspecies differences between mice and pigs, as well as the lack of polymicrobial infection simulation in the present model, the observed efficacy cannot be directly generalized to clinical porcine endometritis management. Further validation in porcine models and more clinically simulated infection systems is required before practical application. Collectively, this study provides a novel non-antibiotic therapeutic candidate and offers a promising antibiotic-alternative strategy for the prevention and treatment of bacterial endometritis in swine breeding. Nevertheless, further systematic pre-clinical verification and porcine clinical trials are still indispensable to validate its safety and translational value before practical swine application.

## Data Availability

The data presented in this study are available on request from the corresponding author due to confidentiality restrictions related to the farm information.

## Supplementary Materials

The following supporting information can be downloaded at: https://www.mdpi.com/article/10.3390/ani16182964/s1, Figure S1: (A) Zeta potentials of Cur@ZIF-8 NPs in water; (B) Hydrodynamic size of Cur@ZIF-8 NPs in water; Figure S2: (A) UV absorption spectra of curcumin; (B) Standard curve of curcumin; Figure S3: Body-weight changes of mice in each group; Table S1: Judgement criteria of antimicrobial susceptibility test (mm); Table S2: Histopathological scoring criteria for the uterus of mice; Table S3: Original Data of Bacterial Isolation and Identification Results; Table S4: Raw inhibition zone diameter measurements (mm) for antimicrobial susceptibility testing of bacterial isolates from porcine endometritis.
