# Supplementary material for: Curcumin Loaded ZIF 8 Nanomaterials Ameliorate Porcine Endometritis Like Lesions in a Mouse Model via Regulating Macrophage Polarization and Antibacterial Activity
Source: Animals (Basel). 2026 Sep 20;16(18):2964. doi: 10.3390/ani16182964 (PMC13603797; doi:10.3390/ani16182964)
Supplement: Supplementary file 1 [file animals-16-02964-s001.zip › animals-4490777-supplementary.pdf]

Electronic Supplementary Information

Curcumin Loaded ZIF 8 Nanomaterials Ameliorate Porcine Endometritis Like Lesions in a Mouse Model via Regulating Macrophage Polarization and Antibacterial Activity

Table S1 Judgement criteria of antimicrobial susceptibility test (mm)

**Antimicrobial categories**	**Antimicrobial Drug**	***E. coli/ P. mirabilis***			***S. aureus***		
		**S**	**I**	**R**	**S**	**I**	**R**
β -lactams	Ampicillin	≥ 17	14 - 16	≤ 13	≥ 29	-	≤ 28
	Cefotaxime	≥ 23	15 - 22	≤ 14	≥ 23	15 - 22	≤ 14
Aminoglycosides	Streptomycin	≥ 15	12 - 14	≤ 11	≥ 15	12 - 14	≤ 11
Tetracyclines	Tetracycline	≥ 15	12 - 14	≤ 11	≥ 15	12 - 14	≤ 11
Quinolones	Enrofloxacin	≥ 23	17 - 22	≤ 16	≥ 23	17 - 22	≤ 16
Chloramphenicol	Florfenicol	≥ 18	13 - 17	≤ 12	≥ 18	13 - 17	≤ 12

Note: R: Resistant; I: Intermediate; S: Sensitive

Table S2 Histopathological scoring criteria for the uterus of mice

Scoring Item	Score 0	Score 1	Score 2	Score 3
Endometrial structural integrity	Intact structure, regularly arranged glands, no rupture or necrosis	Basically intact, mild disorder of a small number of glands, no necrosis	Partially damaged, significantly disordered glands, with a small number of necrotic foci (≤5%)	Severely damaged, massive gland rupture and dissolution, extensive necrosis (>5%)
Inflammatory cell infiltration	No obvious inflammatory cell infiltration, or only scattered individual inflammatory cells	Mild inflammatory cell infiltration, scattered in the endometrial stroma (≤25%)	Moderate inflammatory cell infiltration, focally distributed in the stroma and around glands (26%–50%)	Severe inflammatory cell infiltration, diffusely distributed throughout the entire endometrium (>50%)
Endometrial epithelial injury	Intact epithelium with normal cellular morphology, no exfoliation or degeneration	A small number of epithelial cells with degeneration, no obvious exfoliation	Partial epithelial exfoliation, with obvious degeneration of remaining epithelial cells	Extensive epithelial exfoliation, with only a small number of degenerated cells remaining
Congestion and edema	No obvious congestion or edema, normal vascular morphology	Mild congestion and edema, mild vascular dilation	Moderate congestion and edema, significantly dilated blood vessels, and mild stromal edema	Severe congestion and edema, highly dilated blood vessels, and obvious edematous, loose stroma

Table S3 Original Data of Bacterial Isolation and Identification Results

**Sample**	***P. mirabilis***	***E. coli***	***S. aureus***	***K. pneumoniae***	***E. faecalis***	***Shigella***	***Salmonella***
1	+	-	-	-	+	-	-
2	+	+	-	-	-	-	-
3	+	+	-	-	-	-	-
4	+	-	-	-	-	-	-
5	+	-	+	-	-	-	-
6	+	-	-	-	-	-	-
7	-	-	+	-	-	-	-
8	+	-	-	+	-	-	-
9	+	-	-	-	-	-	-
10	+	-	+	-	-	-	-
11	+	-	-	+	-	-	-
12	+	+	-	-	-	-	-
13	+	+	-	-	-	-	-
14	+	+	-	-	-	-	-
15	+	-	-	-	+	-	-
16	+	-	-	-	-	-	-
17	+	-	-	-	+	-	-
18	+	-	-	-	+	-	-
19	-	-	+	-	-	-	-
20	+	-	-	-	-	-	+
21	-	-	+	-	-	-	-
22	+	+	-	-	-	-	-
23	+	-	-	+	-	-	-
24	+	+	-	-	-	-	-
25	+	-	-	-	-	-	-
26	+	-	-	-	-	-	-
27	+	+	-	-	-	-	-
28	+	+	-	-	-	-	-
29	+	-	-	-	-	-	-
30	+	+	-	-	-	-	-
31	+	-	-	+	-	-	-
32	+	+	-	-	-	-	-
33	+	-	-	-	+	-	-
34	+	+	-	-	-	-	-
35	-	-	+	-	-	-	-
36	-	-	+	-	-	-	-
37	+	+	-	-	-	-	-
38	+	-	-	+	-	-	-
39	+	+	-	-	-	-	-
40	+	-	-	-	-	-	-

Continuation Table S3 Original Data of Bacterial Isolation and Identification Results

Sample	*P. mirabilis*	*E. coli*	*S. aureus*	*K. pneumoniae*	*E. faecalis*	*Shigella*	*Salmonella*
41	+	-	-	-	-	-	-
42	+	+	-	-	-	-	-
43	+	-	-	+	-	-	-
44	+	+	-	-	-	-	-
45	+	-	-	-	-	-	-
46	+	-	-	-	-	-	-
47	+	-	-	-	-	+	-
48	+	+	-	-	-	-	-

Table S4 Raw inhibition zone diameter measurements (mm) for antimicrobial susceptibility testing of bacterial isolates from porcine endometritis

Strain_ID	Species	AMP(mm)	CEF(mm)	STR(mm)	TET(mm)	ENR(mm)	FFC(mm)
PM01	*Proteus mirabilis*	11.2	27.3	10.1	15.6	24.5	12
PM02	*Proteus mirabilis*	10.5	26.8	9.8	15.2	25.1	11.4
PM03	*Proteus mirabilis*	12.1	27.9	8.7	13.8	23.6	15.2
PM04	*Proteus mirabilis*	9.6	26.2	10.6	13.2	26.3	14.7
PM05	*Proteus mirabilis*	8.8	25.7	9.3	12.6	24.8	13.5
PM06	*Proteus mirabilis*	11.5	25.3	8.2	12.1	25.5	11.8
PM07	*Proteus mirabilis*	10.2	24.8	9.5	11.3	23.2	10.6
PM08	*Proteus mirabilis*	9.1	24.2	8.9	10.8	24.1	9.7
PM09	*Proteus mirabilis*	12.3	23.7	10.3	10.2	25.7	11.1
PM10	*Proteus mirabilis*	11	23.3	9.1	9.7	26.6	10.3
PM11	*Proteus mirabilis*	8.5	22.8	8.4	9.2	24.3	9.2
PM12	*Proteus mirabilis*	9.8	22.1	10.7	8.8	23.8	8.6
PM13	*Proteus mirabilis*	10.7	21.5	9.6	8.3	25.2	10.8
PM14	*Proteus mirabilis*	11.8	20.9	8.6	7.9	26.1	9.9
PM15	*Proteus mirabilis*	9.3	20.4	10	7.4	24.6	8.9
PM16	*Proteus mirabilis*	8.2	19.8	9.2	7.1	23.4	11.3
PM17	*Proteus mirabilis*	12	19.3	8.3	6.8	25.8	10.1
PM18	*Proteus mirabilis*	10.4	18.6	10.5	6.4	26.4	9.4
PM19	*Proteus mirabilis*	9.5	18.1	9.4	6.1	24.9	8.5
PM20	*Proteus mirabilis*	11.3	17.5	8.7	5.8	23.9	10.5
PM21	*Proteus mirabilis*	8.9	16.9	9.8	5.4	25.4	9.6
PM22	*Proteus mirabilis*	10.9	16.3	8.1	5.1	26.2	8.8
PM23	*Proteus mirabilis*	9.7	15.8	10.2	4.8	24.4	11
PM24	*Proteus mirabilis*	11.6	15.2	9	4.5	23.5	10
PM25	*Proteus mirabilis*	8.4	14.6	8.5	4.2	25	9.1
PM26	*Proteus mirabilis*	10.1	14.1	9.9	3.9	26.5	8.3
PM27	*Proteus mirabilis*	9	13.5	8.8	3.6	24.7	10.7

Continuation Table S4 Raw inhibition zone diameter measurements (mm) for antimicrobial susceptibility testing of bacterial isolates from porcine endometritis

Strain_ID	Species	AMP(mm)	CEF(mm)	STR(mm)	TET(mm)	ENR(mm)	FFC(mm)
PM28	*Proteus mirabilis*	11.1	12.9	10.4	3.3	23.7	9.8
PM29	*Proteus mirabilis*	8.7	12.3	9.3	3	25.3	8.7
PM30	*Proteus mirabilis*	10.6	11.8	8.2	2.7	26	11.2
PM31	*Proteus mirabilis*	9.2	11.2	9.7	2.4	24.2	10.2
PM32	*Proteus mirabilis*	11.4	10.6	8.4	2.1	23.3	9.3
PM33	*Proteus mirabilis*	8.6	10.1	10.1	1.8	25.6	8.4
PM34	*Proteus mirabilis*	10.3	9.5	8.9	1.5	26.7	10.9
PM35	*Proteus mirabilis*	9.4	8.9	9.6	1.2	24	9.5
PM36	*Proteus mirabilis*	11.7	8.3	8.1	1	23.1	8.2
PM37	*Proteus mirabilis*	8.8	7.8	10.3	0.8	25.9	10.4
PM38	*Proteus mirabilis*	10.8	7.2	9.1	0.6	26.8	9
PM39	*Proteus mirabilis*	9.9	6.6	8.6	0.4	24.5	8.1
PM40	*Proteus mirabilis*	11.9	6.1	9.9	0.2	23.6	11.5
PM41	*Proteus mirabilis*	8.3	5.5	8.5	0.1	25.1	10.6
PM42	*Proteus mirabilis*	10	4.9	9.4	15.8	26.3	9.7
PM43	*Proteus mirabilis*	9.6	4.3	8.8	15.4	18.2	18.3
EC01	*Escherichia coli*	17.4	26.5	15.3	15.7	23.8	18.4
EC02	*Escherichia coli*	17.1	26.1	15.1	15.3	24.2	18.1
EC03	*Escherichia coli*	16.8	25.7	14.6	14.2	24.7	17.3
EC04	*Escherichia coli*	16.3	22.4	14.1	13.4	25.3	16.6
EC05	*Escherichia coli*	15.7	21.8	13.5	12.3	25.9	15.2
EC06	*Escherichia coli*	13.2	21.1	12.8	11.4	23.3	11.7
EC07	*Escherichia coli*	12.6	20.3	12.2	10.6	23.6	10.9
EC08	*Escherichia coli*	11.8	19.6	11.5	9.8	24.1	10.2
EC09	*Escherichia coli*	11.1	18.8	10.7	9.1	24.5	9.4
EC10	*Escherichia coli*	10.4	18	10.1	8.4	25	8.7
EC11	*Escherichia coli*	9.7	17.2	9.4	7.7	25.6	18.7
EC12	*Escherichia coli*	9	16.4	8.8	7	26.1	18.2
EC13	*Escherichia coli*	8.3	15.6	8.1	6.3	18.4	17.1
EC14	*Escherichia coli*	7.6	14.8	7.5	5.6	17.6	16.3
EC15	*Escherichia coli*	6.9	14	6.8	4.9	16.2	10.5
EC16	*Escherichia coli*	6.2	13.2	6.2	4.2	15.3	9.6
EC17	*Escherichia coli*	5.5	12.4	5.5	3.5	14.4	8.8
SA01	*Staphylococcus aureus*	17.3	26.4	15.2	15.5	23.7	18.5
SA02	*Staphylococcus aureus*	17	26	14.8	14.3	24.4	18
SA03	*Staphylococcus aureus*	16.5	24.5	14.2	13.1	25.2	14.8
SA04	*Staphylococcus aureus*	12.8	21.3	13.4	12.2	25.8	11.5
SA05	*Staphylococcus aureus*	12.1	20.1	12.6	11.1	26.3	10.4
SA06	*Staphylococcus aureus*	11.4	18.9	11.7	10	18.1	9.3
SA07	*Staphylococcus aureus*	10.7	17.7	10.8	8.9	17.2	8.2

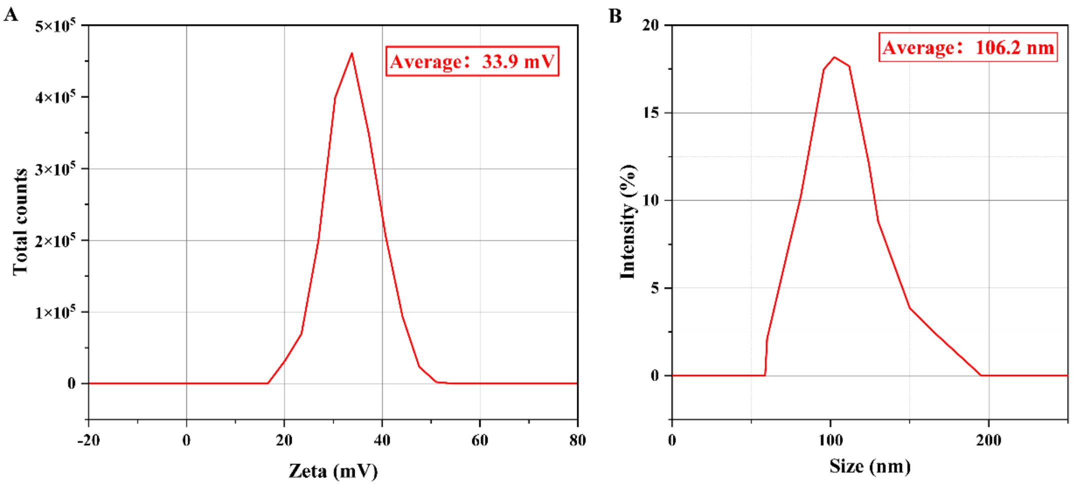

Figure S1 (A) Zeta potentials of Cur@ZIF-8 NPs in water; (B) Hydrodynamic size of Cur@ZIF-8 NPs in water

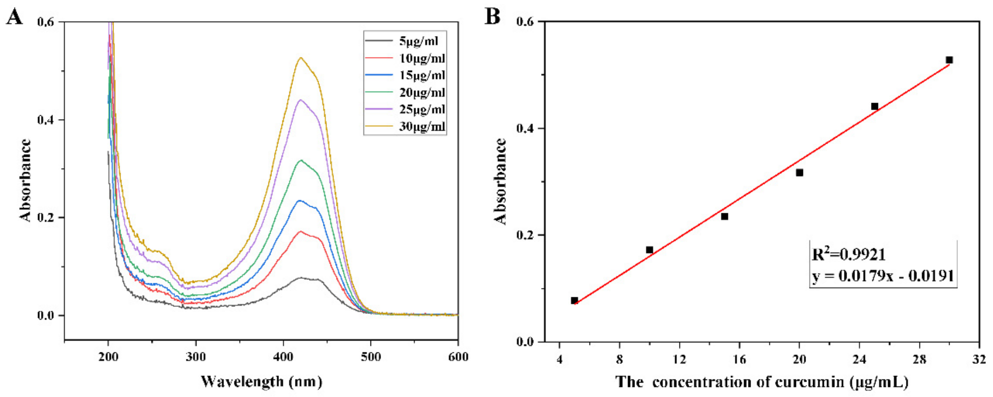

Figure S2 (A) UV absorption spectra of curcumin; (B) Standard curve of curcumin

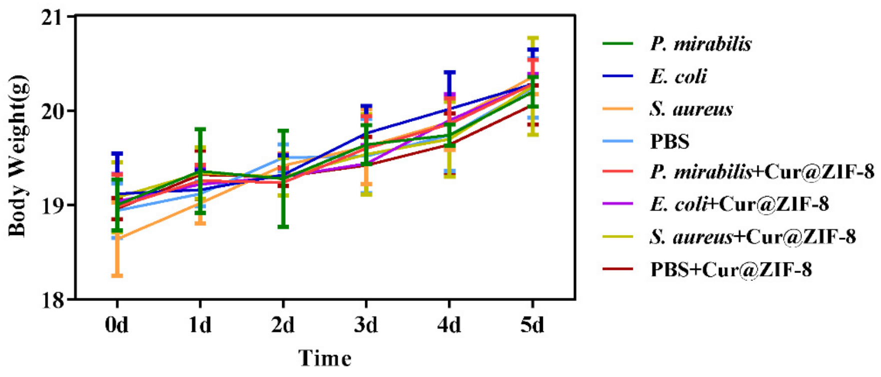

Figure S3 Body-weight changes of mice in each group
